# Gut microbes contribute to variation in solid organ transplant outcomes in mice

**DOI:** 10.1186/s40168-018-0474-8

**Published:** 2018-05-25

**Authors:** Christine M. McIntosh, Luqiu Chen, Alon Shaiber, A. Murat Eren, Maria-Luisa Alegre

**Affiliations:** 1 Department of Medicine, The University of Chicago, Chicago, USA; 2 Marine Biological Laboratory, Woods Hole, USA

**Keywords:** Organ transplantation, Microbiome, Acute allograft rejection, Fecal microbiota transplantation, *Alistipes*

## Abstract

**Background:**

Solid organ transplant recipients show heterogeneity in the occurrence and timing of acute rejection episodes. Understanding the factors responsible for such variability in patient outcomes may lead to improved diagnostic and therapeutic approaches. Rejection kinetics of transplanted organs mainly depends on the extent of genetic disparities between donor and recipient, but a role for environmental factors is emerging. We have recently shown that major alterations of the microbiota following broad-spectrum antibiotics, or use of germ-free animals, promoted longer skin graft survival in mice. Here, we tested whether spontaneous differences in microbial colonization between genetically similar individuals can contribute to variability in graft rejection kinetics.

**Results:**

We compared rejection kinetics of minor mismatched skin grafts in C57BL/6 mice from Jackson Laboratory (Jax) and Taconic Farms (Tac), genetically similar animals colonized by different commensal microbes. Female Tac mice rejected skin grafts from vendor-matched males more quickly than Jax mice. We observed prolonged graft survival in Tac mice when they were exposed to Jax mice microbiome through co-housing or fecal microbiota transplantation (FMT) by gastric gavage. In contrast, exposure to Tac mice did not change graft rejection kinetics in Jax mice, suggesting a dominant suppressive effect of Jax microbiota. High-throughput sequencing of 16S rRNA gene amplicons from Jax and Tac mice fecal samples confirmed a convergence of microbiota composition after cohousing or fecal transfer. Our analysis of amplicon data associated members of a single bacterial genus, *Alistipes*, with prolonged graft survival. Consistent with this finding, members of the genus *Alistipes* were absent in a separate Tac cohort, in which fecal transfer from Jax mice failed to prolong graft survival.

**Conclusions:**

These results demonstrate that differences in resident microbiome in healthy individuals may translate into distinct kinetics of graft rejection, and contribute to interpersonal variability in graft outcomes. The association between *Alistipes* and prolonged skin graft survival in mice suggests that members of this genus might affect host physiology, including at sites distal to the gastrointestinal tract. Overall, these findings allude to a potential therapeutic role for specific gut microbes to promote graft survival through the administration of probiotics, or FMT.

**Electronic supplementary material:**

The online version of this article (10.1186/s40168-018-0474-8) contains supplementary material, which is available to authorized users.

## Background

Solid organ transplantation is a common treatment for end-stage organ failure. However, most transplant recipients need to remain on lifelong immunosuppression to prevent immune-mediated acute rejection of the donor organ, leaving them susceptible to infections [1], malignancies [2], and drug toxicity [3]. In the absence of immunosuppression, the transplant recipient’s immune system recognizes the donor organ as non-self and mounts an immune response, termed the alloimmune response [4]. Even in patients taking immunosuppressive drugs, an alloimmune response can occur and cause acute graft rejection leading to permanent damage to and loss of the transplanted organ. Importantly, patients who are successfully treated for episodes of acute rejection experience worse long-term graft survival than patients who never experience an acute rejection episode [5].

Recipients of solid organ transplants show heterogeneity in the occurrence and timing of acute rejection episodes. While some patients experience acute rejection within the first year after transplantation, others retain their grafts long-term despite similar immunosuppression [6, 7], and a small subset spontaneously develops operational tolerance, the ability to maintain allografts without rejection after withdrawal of immunosuppression [8, 9]. Understanding the factors responsible for such heterogeneity may lead to improved screening protocols for patients and the development of therapeutics to prevent or treat acute rejection.

The likelihood and intensity of acute rejection episodes is mainly determined by the extent of genetic disparities between the donor and the recipient of the allograft. However, a role for environmental factors is emerging. High-fat [10] and high-salt diet [11] have been shown to lead to accelerated transplant rejection in mice. Additionally, infection has been associated with increased incidence of acute rejection in kidney [12, 13] and lung [14, 15] transplant patients. In mice, infection with *Staphylococcus aureus* [16] or *Listeria monocytogenes* [17] prevented the induction of graft-specific tolerance and *L. monocytogenes* infection could also break transplantation tolerance after it had been established [18]. Given its role in the development and function of the immune system [19], and the molecular similarity between pathogens and commensal microbes, the microbiota may also contribute to the intensity of alloimmunity and the kinetics of acute rejection.

Our group has previously shown that germ-free and antibiotic-pre-treated mice exhibit dampened alloimmunity, and prolonged survival of skin grafts, and that gastric inoculation of germ-free mice with FMT from conventional mice is sufficient to accelerate skin graft rejection [20]. These findings indicate that the microbiota is an environmental factor that can causally affect alloimmunity and that massive alterations in the microbiota can translate into measurable differences in graft outcome. However, the comparison between sterile and colonized mice is not representative of the differences in microbiota composition between individuals. In this study, using a murine model of skin transplantation, we set out to determine whether differences in resident microbiota between healthy individuals at steady state may impact graft survival.

Investigating communities of commensal microbes associated with different skin transplant outcomes may allow identification of specific members of the microbiota contributing to transplant rejection. Individual microbial taxa have been shown to promote varied immune phenotypes in their hosts. For example, *Bacteroides fragilis* and some *Clostridium* species have been shown to promote the development of regulatory T cells (T_REGS_) [21, 22], and segmented filamentous bacteria have been shown to promote differentiation of naïve T cells into T helper 17 (T_H_17) cells [23] in the gut mucosa. With the recent discovery of specific microbes modulating anti-tumor immunity [24–26], susceptibility to rheumatoid arthritis [27, 28], experimental autoimmune encephalomyelitis [22], and other immune-mediated diseases distal to the gut in mouse models [29], we sought to identify members of the gut microbiota associated with improved or worsened transplant survival. Here, we provide evidence that members of the genus *Alistipes* are associated with improved survival of transplanted skin in mice.

## Results

### Microbiota composition differs in Jax and Tac mice of the same genetic background

To determine whether specific commensal microbes could impact skin transplantation outcomes, we obtained mice of the same C57BL/6 (B6) genetic background from different vendors as they have been shown to be colonized by different communities of commensal microbes [23, 24]. We performed 16S rRNA gene amplicon sequencing of fecal samples from Jax and Tac mice upon their arrival to our facility. We inferred highly resolved microbial community structures in our amplicon data with Minimum Entropy Decomposition (MED). MED iteratively decomposes a given amplicon dataset using highly variable nucleotide positions identified by Shannon entropy, until the variation within the population of amplicon sequences that resolve to the same oligotype, or “amplicon sequence variant” (ASV), is minimal [30]. This strategy allows the identification of closely related but distinct taxa at a single-nucleotide resolution, better explaining micro-diversity compared to clustering strategies that rely on arbitrary similarity thresholds [31–34].

**Fig. 1:**
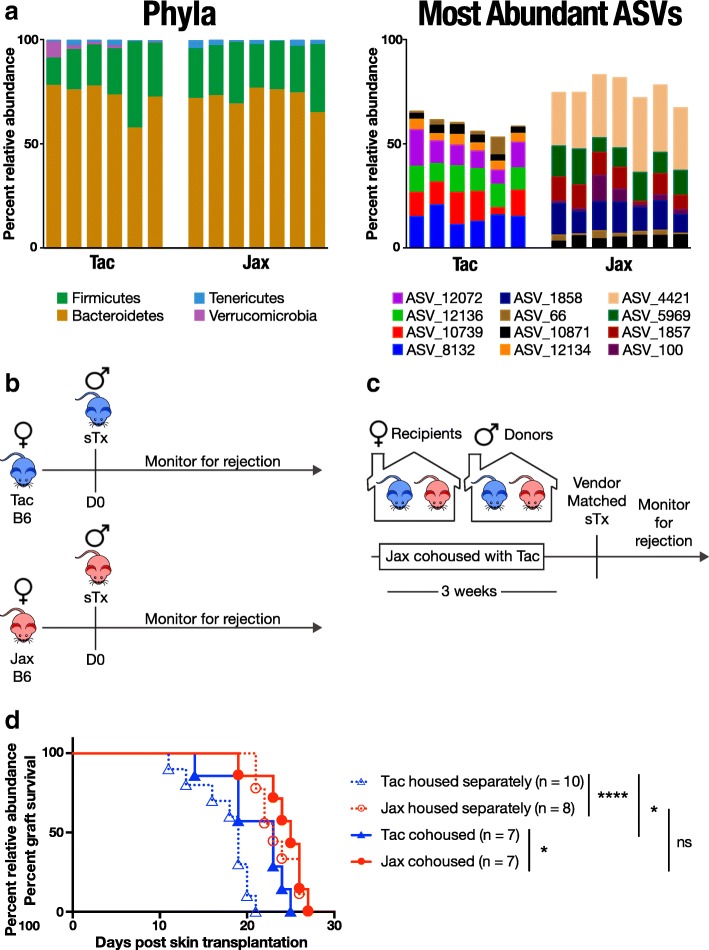
Cohousing with Jax B6 mice results in prolonged skin transplant survival in Tac B6 mice. **a** Representation of phyla (left) or the 12 ASVs with highest average percent relative abundance across pre-cohousing Jax and Tac samples (right) after 16S rRNA gene sequencing and clustering of amplicons into ASVs using MED in female Tac and Jax B6 mice. Each column represents an individual mouse. All phyla contributing to at least 1% of overall microbial composition are labeled and shown in color. **b**–**d** Survival of vendor-matched male B6 skin transplants in female Tac and Jax B6 mice housed separately or following cohousing of Jax and Tac skin graft donors and cohousing of Jax and Tac recipients for 3 weeks prior to transplantation. *P < 0.05, ****P < 0.0001; ns = not significant

MED identified 195 ASVs present in female Jax or Tac mice (Additional file 1). Female Jax and Tac mice were both colonized almost entirely by members of the phyla *Firmicutes* and *Bacteroidetes* and a small proportion of the phylum *Tenericutes* (Fig. 1a left, Additional file 2), which agrees with previous studies [24]. Tac mice were also colonized by a small proportion of ASVs assigned to the phylum *Verrucomicrobia*. While these ASVs made up only 2.1% of the Tac mice microbiota on average, this was a relative abundance 700-fold greater than in Jax mice. This lack of *Verrucomicrobia* members in Jax mice is consistent with our previous findings [24].

To gain an understanding of the differences between Jax and Tac microbiota composition beyond taxonomic annotations, we compared the most abundant ASVs in Jax and Tac mice that made up at least 50% of the overall composition of each mouse (Fig. 1a right, Additional file 3). The distribution of the 12 ASVs that matched this criterion differed remarkably between Jax and Tac mice except two. One of the two ASVs that were similarly abundant in both groups (ASV_10871) could not be assigned taxonomy beyond the order *Bacteroidales*, while the other (ASV_66) resolved to the genus *Lactobacillus*. Five of the remaining ten ASVs (8132, 10739, 12136, 12072 and 12134) were specific to Tac mice, as they were detected in every Tac mouse while being virtually undetected in Jax mice. None of the Tac-specific ASVs could be assigned taxonomy at a level higher than *Bacteroidales* order. Remaining five ASVs were specific to Jax mice, three of which (ASVs 1857, 1858, and 4421) were not assigned beyond the order *Bacteroidales*, and the last two were assigned to *Allobaculum (*ASV_100) and *Alistipes* (ASV_5969). Some lower abundance ASVs were also differentially abundant between Jax and Tac mice (Additional file 3). Relative abundance of ASVs_122, 5039, and 5968 were all over 1000-fold higher in Tac than in Jax mice and were assigned to family *Prevotellaceae*, genus *Lactobacillus*, and genus *Parabacteroides*, respectively. Conversely, the *Bacteroidales*-assigned ASV_1859 was over 1000-fold more abundant in Jax than in Tac.

### Skin graft rejection kinetics in Tac mice are affected by cohousing or FMT

Following our observation of differences in microbiota composition between Jax and Tac mice arriving at our facility, we compared the kinetics of rejection of skin grafts bearing sex-dependent minor (non-major histocompatibility complex) mismatches, as skin from male mice expresses H-Y antigens encoded by the Y chromosome [35, 36]. H-Y antigens are recognized following transplantation by the immune system of otherwise genetically identical female recipients, resulting in rejection. We grafted skin from male B6 Jax and Tac mice to their female B6 counterparts from the same vendor (Fig. 1b) and monitored animals to determine the length of time until each graft was rejected (Fig. 1d). Female Tac mice rejected male skin grafts significantly faster (MST 18 ± 3 days, *n* = 10) than did female Jax mice (MST 24 ± 2 days, *n* = 8; *P* < 0.001). To eliminate the possibility that genetic drift in B6 mice at Jax or Tac facilities had contributed to the observed differences in rejection kinetics, we cohoused Jax and Tac skin graft donor mice together for 3 weeks prior to transplantation, and Jax and Tac skin graft recipients together for 3 weeks prior to transplantation (Fig. 1c), to allow transfer of some environmental factors, including commensal microbes, between individuals from different vendors, while preserving any existing host genetic differences. In support of an environmental but not host genetic cause for the delayed rejection in Jax mice, Tac mice rejected grafts with slower Jax-like kinetics after cohousing (Fig. 1d) (MST 21 ± 4 days, *n* = 7; *P* = 0.02 when compared to Tac mice housed separately from Jax mice). In contrast, rejection kinetics in Jax mice remained unchanged (MST 24 ± 3 days, *n* = 7) when compared with Jax mice housed separately from Tac mice, suggesting that Jax mice were transferring a dominant inhibitory factor to Tac mice by cohousing, rather than Jax mice losing a graft-protective factor or gaining a rejection-accelerating factor by cohousing with Tac mice.

**Fig. 2:**
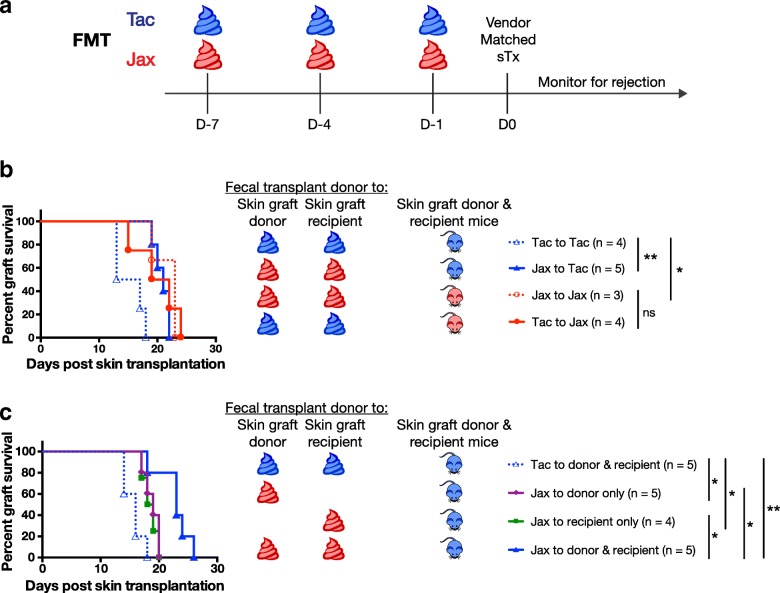
Jax FMT into both Tac skin transplant donor and recipient mice delays skin transplant rejection. **a** Treatment schedule for mice receiving Jax or Tac FMT. **b** Survival kinetics of vendor-matched male B6 skin transplants in female Jax or Tac B6 graft recipients following Jax or Tac FMT into both skin transplant donor and recipient. **c** Survival of male Tac B6 skin transplants in female Tac B6 recipients following Jax or Tac FMT into skin transplant donor and/or recipient. *P < 0.05, **P < 0.01; ns = not significant

Because components of the fecal microbiota are transferred between cohoused mice, we sought to determine whether the environmental factor responsible for differences in skin transplantation outcomes in Jax mice could be found in Jax feces. To this end, skin graft donor and skin graft recipient Tac mice received gastric FMT from Jax mice, prior to skin transplantation (Fig. 2a). Indeed, Jax FMT into Tac mice promoted slower skin graft rejection by Tac mice when compared to Tac skin graft recipients that had received Tac FMT (MST 21 ± 1 days, *n* = 5 versus 15 ± 3 days, *n* = 4; *P* < 0.005) (Fig 2b). In contrast, Tac or Jax FMT into Jax mice did not change graft rejection kinetics in Jax mice (MST 20 ± 4 days, *n* = 4 versus MST 22 ± 2 days, *n* = 3). This confirmed the presence of a component of Jax feces that dominantly prolonged skin graft survival and could be transferred to Tac mice by FMT.

### Jax FMT to both Tac skin graft donor and Tac skin graft recipient is required for maximal prolongation of graft survival

In organ transplantation, genetic or environmental factors associated with either the donor or the recipient could influence the rejection of the transplanted organ. For instance, polymorphisms of both the transplant donor [37, 38] and recipient [39–41] have been shown to impact graft rejection, and we have previously demonstrated that antibiotic pre-treatment needs to be administered to both the donor and the recipient to result in prolonged skin graft survival [20]. In the cohousing and fecal transfer experiments described in Figs. 1 and 2, skin donors and recipients were both cohoused or given FMT prior to transplantation, so the effect of Jax feces on graft survival could have occurred through changes to the donor and/or recipient. To determine whether the improvement in skin transplantation outcome in Tac mice after exposure to Jax microbiota occurred through changes to the donor, recipient, or both, only Tac skin graft donors or recipients received FMT from Jax mice prior to transplantation (Fig. 2c). While Jax FMT into only the Tac skin graft donor (MST 19 ± 1 days, *n* = 5) or only the Tac skin graft recipient (MST 19 ± 1 days, *n* = 4) led to a slight improvement in skin transplant survival compared to Tac FMT into Tac mice (MST 16 ± 2 days, *n* = 5), Jax FMT into both Tac skin graft donor and recipient led to more significant prolongation in graft survival (MST 23 ± 3 days, *n* = 5; *P* < 0.01) when compared to Tac FMT. Thus, maximal skin graft survival improvement by Jax FMT required that both the Tac skin graft donor and the Tac skin graft recipient receive the Jax FMT.

### Convergence of fecal microbiota composition in Jax and Tac mice following cohousing and FMT

**Fig. 3:**
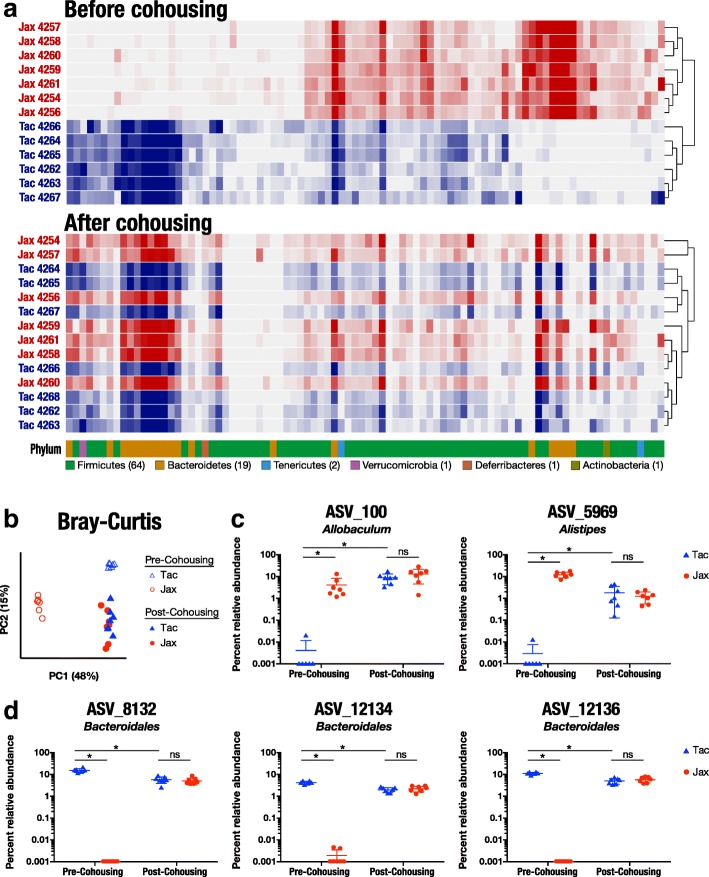
Jax and Tac microbiota composition converges following cohousing. **a** Heat map of percent relative abundance of ASVs in Jax and Tac mice before and after cohousing. Each row represents an individual mouse. Each column represents a single ASV. Only ASVs with percent relative abundance of at least 1% in a minimum of two samples from the same vendor before cohousing were included. Arrangement of ASVs was kept constant between all rows. Bottom row displays the phylum to which the ASV in the same column was assigned. **b** Principal coordinate analysis of female B6 Jax and Tac fecal microbiota composition using Bray-Curtis distance prior to or following cohousing of Jax and Tac mice for 3 weeks. Each dot represents an individual mouse. **c**, **d** Percent relative abundance of all ASVs significantly differentially abundant between Tac and Jax before cohousing and between Tac before and after cohousing with Jax, but not significantly different between Jax and Tac after cohousing. Each dot represents an individual mouse. *P < 0.05, ns = not significant

**Fig. 4:**
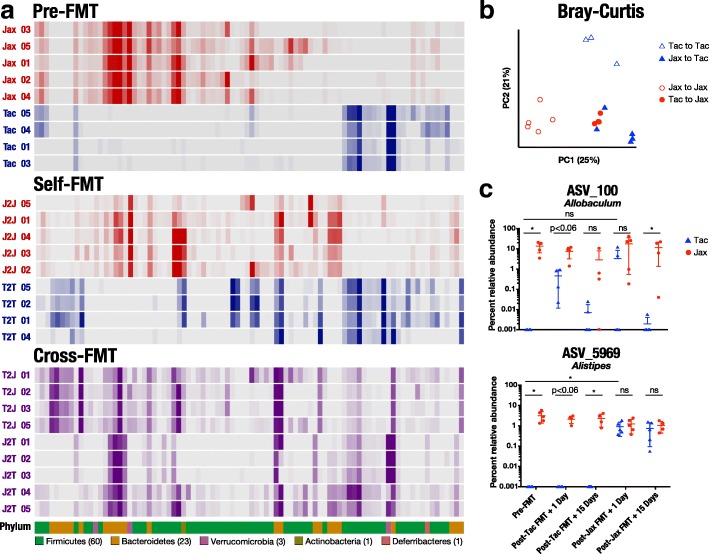
Jax and Tac microbiota composition converges following FMT, including transfer of ASVs transferred by cohousing. **a** Heat map of percent relative abundance of ASVs in Jax and Tac mice before FMT, or 1 day after the third FMT from either vendor-matched fecal donor (self-FMT) or fecal donor from different vendor (cross-FMT). Each row represents an individual mouse. Each column represents a single ASV. Only ASVs with percent relative abundance of at least 1% in a minimum of two samples from the same vendor were included. Arrangement of ASVs was kept constant between all rows. Bottom row displays the phylum to which the ASV in the same column was assigned. **b** Principal coordinate analysis of female B6 Jax and Tac fecal microbiota composition using Bray-Curtis distance following either self-FMT (Jax to Jax, Tac to Tac) or cross-FMT (Tac to Jax, Jax to Tac). **c** Percent relative abundance of ASVs associated with prolonged graft survival in Jax and Tac mice either pre-FMT, or 1 or 15 days after the third self-FMT or cross-FMT. Each dot represents an individual mouse. *P < 0.05, ns = not significant

To determine whether Jax fecal transfer led to changes in the microbiota of Tac mice, we compared microbial community profiles in samples from Jax and Tac mice before and after cohousing or FMT experiments. The distinct composition of Jax and Tac microbiota composition at baseline and their convergence after cohousing or cross-vendor FMT was clearly evident in the relative abundance of microbes at the ASV level (Figs. 3a and 4a). Principal coordinate analysis (PCoA) of these data also revealed a clear separation of Jax (*n* = 7) and Tac (*n* = 6) fecal samples at baseline and a convergence of Jax (*n* = 7) and Tac (*n* = 7) microbiota composition following cohousing (Fig. 3b). PCoA showed a separation between Jax and Tac mice following FMT from vendor-matched fecal donors, but similar to cohousing, cross-vendor-FMT resulted in a convergence of microbiota composition (Fig. 4b). Alpha diversity as defined by Shannon entropy or inverse Simpson index was similar in Jax and Tac fecal samples, and longer graft survival did not correlate with differences in alpha diversity (Additional file 4). These results suggest that one or more taxa are transferred from Jax to Tac by cohousing or FMT and that the transfer is associated with prolonged skin graft survival in Tac mice.

We then analyzed the microbial community structure between these cohorts to determine which taxa changed in Tac mice following cohousing with Jax mice or following Jax FMT that could explain the improvement in rejection kinetics in Tac mice. To gain a broad view of the efficiency of microbial transfer in our experiments, we determined the number of ASVs detected in the majority of mice from either Jax or Tac prior to cohousing or FMT and quantified how many of these ASVs were gained or lost post cohousing or FMT (Additional file 5). Of 30 Jax-specific ASVs, 14 were gained by Tac mice post cohousing and 21 out of 27 Tac-specific ASVs were gained by Jax post cohousing. Fourteen of 60 Jax-specific ASVs were transferred to Tac by Jax FMT, and 8 of 15 Tac-specific ASVs were transferred to Jax by Tac FMT. Five ASVs were gained by Tac mice by both cohousing and Jax FMT. Three ASVs were gained by Jax mice by both cohousing and Tac FMT.

We chose to focus further analysis on ASVs that had a relative abundance of at least 1% in a minimum of two mice from either Jax or Tac at baseline, reasoning that very low abundance microbes were more likely to represent sequencing errors. Of the 34 ASVs meeting our criteria for inclusion, our analysis revealed 24 ASVs that were differentially abundant between Jax and Tac mice upon arrival at our facility (Additional file 6). Following cohousing, none of those 24 ASVs remained significantly different between Jax and Tac, and the relative abundance of only five (ASVs_3100, 5969, 8132, 12134, and 12135) had changed in Tac mice before and after cohousing with Jax (Fig. 3c, d). ASVs_100 and ASV_5969 were present only in Jax mice at baseline and were transferred to Tac mice by cohousing, while remaining unchanged in Jax mice. Three ASVs that resolved to the *Bacteroidales* order (ASVs_8132, 12134, and 12136) were present only in Tac mice at baseline, and their abundance increased significantly in Jax mice after cohousing. They also decreased two to threefold in relative abundance in Tac mice after cohousing, though the increase in these ASVs in Jax mice was far greater than their decrease in Tac mice. Because ASVs_100 and ASV_5969 only changed in Tac following cohousing, we reasoned that the abundance of these ASVs tracked best with delayed skin transplant rejection and that these were the best candidates for ASVs promoting skin transplant survival.

Next, we analyzed taxonomic composition of the fecal microbiota in Jax and Tac mice prior to and following Jax or Tac FMT (Additional file 7). ASVs_100 and ASV_5969 were again detected in Jax (*n* = 5) but not Tac (*n* = 4) mice upon arrival at our facility (Fig. 4c). Following Jax FMT into Tac (*n* = 5), relative abundance of ASV_100 rose in two mice to match relative abundance in Jax mice but returned to nearly undetectable levels within 15 days after FMT. Tac mice gavaged with Tac feces (*n* = 4) also acquired some ASV_100. Jax mice maintained relatively high levels of ASV_100 whether gavaged with Jax or Tac feces, though the relative abundance did drop in some Jax mice. Much better correlated with graft outcome was ASV_5969, which was consistently transferred to Tac mice given Jax FMT, reaching levels similar to those seen in Jax mice. Relative abundance of ASV_5969 remained consistently high 15 days following the last FMT, showing that colonization was stable over time and through the stress of surgery. Jax mice maintained consistently high levels of ASV_5969 whether given FMT from Jax (*n* = 5) or Tac (*n* = 4) mice. We also note that, slower or faster skin graft rejection was associated with the presence or absence of ASV_100 and ASV_5969, independent of their relative abundance in animals in which they were detected (data not shown).

### ASV_100 and ASV_5969 were absent from an outlier cohort where Jax FMT did not prolong skin transplant survival in Tac mice

**Fig. 5:**
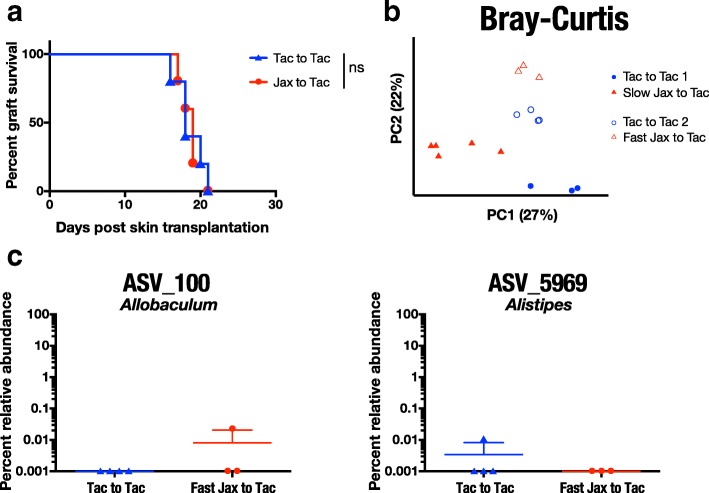
Skin transplant survival-associated ASVs are missing from cohort of Jax to Tac mice with fast rejection. **a** Male B6 Tac skin transplant survival kinetics in an outlier cohort of female B6 Tac mice that had received Jax or Tac FMT. **b** Principal coordinate analysis of microbiota composition using Bray-Curtis distance in female Tac B6 mice treated with Tac FMT (Tac to Tac 1 and Tac to Tac 2), with Jax FMT that delayed graft rejection (Slow Jax to Tac) or Jax FMT that did not delay graft rejection (Fast Jax to Tac). Each dot represents an individual mouse. **c** Percent relative abundance of ASVs previously associated with prolonged graft survival (ASV_100 and ASV_5969) in Tac mice shown in **a** 1 day after the third Tac or Fast Jax FMT. ns = not significant

Importantly, in one experiment, Tac mice treated with Jax FMT did not display delayed rejection kinetics (MST 19 ± 2 days, *n* = 5) when compared with Tac FMT-treated Tac mice (MST 19 ± 1 days, *n* = 5) (Fig. 5a), affording us the opportunity to compare microbiota composition between these Jax FMT-treated Tac mice with fast rejection kinetics (fast Jax to Tac) and Jax FMT-treated Tac mice with delayed graft rejection from prior experiments (slow Jax to Tac). We performed a PCoA of 16S rRNA gene sequencing data in fecal DNA isolates from Tac FMT-treated Tac mice (Tac to Tac 1, *n* = 3 and Tac to Tac 2, *n* = 4), slow Jax FMT-gavaged Tac mice (*n* = 5), and fast Jax FMT-gavaged Tac mice (*n* = 3) using Bray-Curtis distance. Our analysis revealed that mice exhibiting delayed skin graft rejection (slow Jax FMT-gavaged Tac mice) clustered separately from mice rejecting grafts more quickly (Tac mice and fast-Jax FMT-treated Tac mice) (Fig. 5b), supporting our hypothesis that microbiota composition impacts graft rejection kinetics. Consistent with a role for ASV_100 or ASV_5969 in prolonging skin graft survival, fast Jax FMT-treated Tac mice were nearly devoid of both of these ASVs (*n* = 2, Fig. 5c, Additional file 8).

## Discussion

In this study, we have determined that the composition of the fecal microbiota impacts the kinetics of skin transplant rejection in otherwise unmanipulated mice (i.e., not treated with antibiotics nor immunosuppressed). In the human population, there is a large diversity of microbiota composition between individuals, variability which can already be observed within the first year of life [42]. Even within an individual, large shifts in microbiota composition can occur through time following dietary changes [42, 43]. Our findings show that such differences in microbial community composition between individuals at steady state, independent of genetic differences between graft donor and recipient, can translate into varied kinetics of graft rejection and may therefore contribute to inter-patient variability in transplant outcomes.

We have identified a specific bacterial group that resolved to the genus *Alistipes* (ASV_5969), whose presence consistently correlated with delayed skin graft rejection in Jax mice or in Tac mice following cohousing with Jax mice or following Jax FMT pre-treatment. While transfer of ASV_5969 from Jax to Tac was consistently associated with prolonged graft survival, the mechanism by which members of this ASV could provide such an effect remains uncertain. Prolongation of skin graft survival could occur through directly or indirectly dampened adaptive alloimmunity, improved wound healing, or other processes impacting longevity of a transplanted organ. Alternatively, ASV_5969 may be displacing, after transfer into Tac mice, microbial species that promote faster graft rejection. Such displacement would need to be broadly distributed across different genera and/or variable between individuals as we did not detect any microbial taxon consistently displaying a negative correlation with skin transplant survival. Members of ASV_5969 may also inhibit certain behaviors of other commensal microbes that promote graft rejection without limiting their colonization.

Interestingly, optimal prolongation of skin transplant survival in Tac mice required treating both the skin transplant donor and the skin transplant recipient with Jax FMT. As the female skin transplant recipient only receives skin from the male skin transplant donor, and the graft is disinfected with ethanol prior to transplantation, this finding suggests that the effect of Jax FMT in the transplant donor is localized to the skin tissue itself. The skin tissue may be affected, before its harvest for transplantation, by soluble factors circulating from the intestine to the skin, or by direct contact with the modified feces in the cage. Alternatively, we cannot eliminate the possibility that some Jax-derived microbes resist skin disinfection and modulate the immune system of the skin graft host after transplantation. Nevertheless, despite differences in microbiota between male and female mice evident in our data as well as in previous reports [44], ASV_5969 was transferred from Jax to Tac in both male skin graft donors and female skin graft recipients (Additional files 9 and 10), supporting the hypothesis that the same immune modulation is at play in the graft donor and graft recipient.

While mechanisms behind these observations warrant further study, our data suggest a potential immunomodulatory role for the *Alistipes* population identified by ASV_5969. Not much is known about the immune impact of individual members of the *Alistipes* genus. One species, *Alistipes shahii*, has been associated with increased, rather than diminished, immune responses, as it has been shown to improve efficacy of anti-tumor immunotherapy in a mouse model [26]. This effect was attributed to lipopolysaccharide (LPS) on the surface of *A. shahii* acting as a TLR4 ligand, promoting tumor necrosis factor production and thus enhanced anti-tumor immunity. It is unlikely that *Alistipes* is promoting graft survival through LPS-TLR4 interactions, as it is polymorphisms associated with loss rather than gain of function in TLR4 that have been shown to be protective from acute rejection in humans [45, 46], and signaling through TLR4 has been shown to promote rejection in a mouse skin graft model [47].

An alloimmunity-dampening mechanism more consistent with our findings relates to the recent report that *Alistipes* members produce the anti-inflammatory metabolite sulfobacin B [48]. In vitro, incubation of macrophages with sulfobacin B has been shown to inhibit production of tumor necrosis factor (TNF) and nuclear translocation of nuclear factor kappa-light-chain-enhancer of activated B cells (NF-κB) following stimulation with lipopolysaccharide (LPS) [49]. In vivo, intraperitoneal injection with sulfobacin A lessened the inflammatory response to phorbol 12-myristate 13-acetate and LPS. In our *Alistipes*-colonized transplant recipients, it is conceivable that sulfobacin B might similarly impair NF-κB signaling and TNF production, leading to a reduction in the strength of the alloimmune response and thus delayed allograft rejection. Consistent with this, in preliminary results, we found reduced expression of TNF transcripts in the lymph nodes of skin transplanted Jax mice when compared to Tac mice.

While graft survival was only prolonged by several days in our slow-rejecting experimental groups, this modest survival increase may be biologically relevant. Indeed, we and our collaborators previously found similar differences in kinetics of tumor growth in mice obtained from different vendors and differences in tumor control were significantly amplified when the animals were treated with anti-PDL1, a checkpoint blockade therapy [24]. We have since extended these data to the clinical setting and confirmed that differences in the fecal microbiota observed prior to initiation of checkpoint blockade therapy could predict responsiveness to subsequent immunotherapy in melanoma patients [50]. These findings as well as a recent study showing synergy of low-dose tacrolimus with FMT from mice treated with high-dose tacrolimus in delaying the rejection of major mismatched skin allografts in mice [51] suggest that small effects of the microbiota at steady state on tumor control or graft survival may be amplified by pharmacologic treatments. While our study has utilized a minor mismatch model of skin transplant rejection to provide a better window to observe either acceleration or delay in rejection kinetics without requiring immunosuppression, we have recently shown that antibiotic pre-treatment of skin graft donors and recipients can prolong survival of major mismatched skin grafts as well, supporting a wider role of the microbiota in various transplantation settings [20].

The shift in graft rejection kinetics observed between Tac mice treated with slow Jax-FMT and Tac mice treated with fast Jax-FMT, despite the Jax fecal donors originating from the same room in the same breeding facility at Jackson Laboratories, should provide a cautionary tale to investigators, as changes in microbial community composition that may occur over periods of time can impact the phenotype of interest.

One of the most noteworthy outcomes of our study is that there may be microbial populations that consistently colonize newly exposed mice and prolong graft survival. This observation suggests that whether patients undergoing organ transplantation may benefit from probiotic therapies or FMT warrants further research.

Our results also highlight the importance of understanding the effects of surgery and medications given to transplant recipients on microbiota composition, and the potential for them to induce colonization by microbes promoting or impairing graft survival. Studies have shown changes to the microbiota of liver transplant recipients following transplantation, though no changes to relative abundance of *Alistipes* were observed [52]. While the cause of microbiota changes in transplant recipients remains unknown, a recent study has shown that between multiple immunosuppressive drugs commonly used in transplantation, only steroids significantly impacted the composition of the mouse fecal microbiota, including a reduction in *Bacteroidetes* and an increase in *Firmicutes* [53]. Whether these post-transplantation changes to the microbiota have a meaningful impact on transplant outcomes remains to be investigated.

## Conclusions

Our comparison of skin transplant rejection kinetics in mice of the same genetic background from different commercial vendors demonstrated that the composition of the resident microbiome in healthy individuals can impact the kinetics of transplant rejection, and may thus also contribute to inter-individual variability in graft outcomes in patients. We identified a single ASV that was consistently and stably transferred from the fecal microbiota of Jax to Tac and which was associated with prolonged skin graft survival in Tac mice. These observations suggest that we need to better understand transplantation-associated changes to patient microbiota composition as these changes may impact transplant outcomes.

## Methods

### Mice

We obtained C57BL/6 (B6) from Jackson Laboratory or Taconic Farms and performed all experiments using 6–8-week-old male or female mice. We fed mice with Harlan Teklad 2018 diet, maintained them on distilled water, and housed them in individually ventilated cages in a specific pathogen-free animal facility at the University of Chicago. We collected fecal samples directly into autoclaved tubes, avoiding contact with mouse skin or urine, and stored them at − 20 °C until DNA isolation.

### Skin transplantation

We transplanted tail skin from male B6 mice onto the flank of female B6 recipients as previously described [54] and removed bandages after 7 days, while monitoring the graft survival every other day thereafter. We reported “rejection” when less than 20% of transplanted skin was viable.

### Cohousing and FMT experiments

For cohousing experiments, we kept two or three B6 mice from each Jax and Tac cohort in the same cage (five mice/cage) for 3 weeks prior to transplantation. Cohousing experiments prior to transplantation included both skin transplant donors and recipients grouped based on their gender. For FMT experiments, male mice received oral FMT from male mice, while female mice received oral FMT from female mice. FMT donor mice arrived from Jax or Tac in the same shipments as FMT recipient mice, but were housed separately from FMT recipients. We prepared a separate fecal suspension from each of three FMT donor mice by suspending one fecal pellet in 1 mL sterile phosphate buffered saline using a syringe and 18-gauge needle (Becton Dickinson) until all volume could pass through the needle. We allowed particulates to settle for 30 s before drawing supernatant into a syringe attached to a 22-gauge gavage needle (Fine Science Tools). We pooled together 500 μL of the supernatants from each of the three 1 mL suspensions and deposited 200 μL of this pooled supernatant into the stomach of each recipient mouse by gavage. Each FMT recipient was gavaged 7, 4, and 1 days prior to skin transplantation, with donor feces freshly harvested from the same three Jax or Tac FMT donors immediately prior to gavage, and the first FMT obtained upon arrival of the Jax or Tac mice to our facility. We cleaned gavage needles with 70% ethanol between mice, using different gavage needles for each treatment group. We cleaned gavage needles with 70% ethanol and autoclaved them after the final gavage on each experimental day.

### Microbial DNA isolation, sequencing library preparation, and analysis

QIAamp DNA Stool Mini Kit (QIAGEN) extracted the DNA from fecal samples homogenized with 0.1 mm zirconia/silica beads in 1.4 mL ASL buffer (QIAGEN) in a Mini-Beadbeater (Biospec). The Environmental Sample Preparation and Sequencing Facility at Argonne National Laboratory (Argonne, IL, USA) performed library preparation and sequencing of our DNA isolates. Thirty-five cycles of amplification were performed using primers [55] that target the V4-V5 region of the 16S rRNA gene to generate our amplicons from purified DNA, and Illumina MiSeq paired-end sequencing (2 × 300) was used to sequence our amplicon libraries at the High-Throughput Genome Analysis Core in the Argonne National Laboratory. Quantitative Insights Into Microbial Ecology (QIIME) software [56] joined paired-end sequences and de-multiplexed raw sequencing data into samples. We identified ASVs in our dataset to infer high-resolution microbial community structures using Minimum Entropy Decomposition [30] (MED) via the previously described oligotyping pipeline [57]. We used Global Alignment for Sequence Taxonomy (GAST) [58] to infer the taxonomic affiliation of each ASV. After identifying ASVs, QIIME calculated alpha diversity estimates with Shannon entropy and inverse Simpson index. We performed principal coordinate analysis (PCoA) to infer similarities between samples based on their microbial community structures using the relative abundances of ASVs and the Bray-Curtis distance with the R package phyloseq [59] after removing samples with less than 2500 reads. We generated heat map visualizations of ASV percent relative abundances using anvi’o v3 [60].

### Statistical analysis

Kaplan-Meier plots and log-rank (Mantel-Cox) tests compared graft survival curves, and unpaired Mann-Whitney tests with Sidak-Bonferroni correction compared the relative abundance of microbial taxa across samples. We used Prism 6 (Graphpad) for statistical analyses and considered *P* < 0.05 to be statistically significant denoting levels of significance with increasing numbers of asterisks in our text and figures: *P < 0.05, **P < 0.01, ***P < 0.001, ****P < 0.0001.

## Additional files

Additional file 1: ASV counts and number of ASVs detected in all samples. Tables providing total read counts, read counts for each ASV with assigned taxonomy, and the number of ASVs detected for each sample. (XLSX 119 kb)

Additional file 2: Phylum-level fecal microbiota composition in Jax and Tac mice at baseline. Table providing percent relative abundance of all detected phyla in Jax and Tac fecal samples at baseline. (XLSX 10 kb)

Additional file 3: Fecal microbiota ASVs in Jax and Tac mice at baseline. Table providing percent relative abundance of all ASVs and their assigned taxonomy in Jax and Tac fecal samples at baseline and the 12 most abundant ASVs when averaged across all samples. Some ASVs listed were not detected in these samples but were detected in other samples from this study. (XLSX 52 kb)

Additional file 4: Alpha diversity of the fecal microbiota is not associated with prolonged skin transplant survival in Jax mice. Figure showing Shannon entropy and inverse Simpson index of fecal microbiota from female Jax and Tac skin transplant recipients A. before and after cohousing, and B. before and after FMT from male Jax or Tac mice. (PDF 788 kb)

Additional file 5: Transfer efficiency of ASVs following cohousing or FMT. Tables identifying ASVs detected in the majority of mice from either Jax or Tac prior to cohousing or FMT and which of these ASVs were gained or lost by cohousing or FMT. (XLSX 233 kb)

Additional file 6: Fecal microbiota ASVs in Jax and Tac mice before and after cohousing. Tables providing percent relative abundance of all ASVs and their assigned taxonomy in Jax and Tac fecal samples before and after cohousing with results of statistical analysis. Some ASVs listed were not detected in these samples but were detected in other samples from this study. (XLSX 100 kb)

Additional file 7: Fecal microbiota ASVs in Jax and Tac mice before and after FMT. Tables providing percent relative abundance of all ASVs and their assigned taxonomy in Jax and Tac fecal samples before FMT and after self-FMT or cross-vendor-FMT with results of statistical analysis. Some ASVs listed were not detected in these samples but were detected in other samples from this study. (XLSX 79 kb)

Additional file 8: Fecal microbiota ASVs in outlier Tac cohort where Jax FMT did not improve graft survival. Table providing percent relative abundance of all ASVs and their assigned taxonomy and results of statistical analysis in Tac mice following Jax or Tac FMT in a cohort where Jax FMT did not prolong graft survival. Some ASVs listed were not detected in these samples but were detected in other samples from this study. (XLSX 27 kb)

Additional file 9: Fecal microbiota ASVs in male skin transplant donors. Table providing percent relative abundance of all ASVs and their assigned taxonomy in all male skin transplant donors used in this study. Some ASVs listed were not detected in these samples but were detected in other samples from this study. (XLSX 33 kb)

Additional file 10: Relative abundance of ASV_5969 in skin transplant donors is associated with prolonged skin transplant survival. Figure showing percent relative abundance of transplant survival-associated ASVs in male Jax and Tac skin transplant donors A. before and after cohousing, B. before and after FMT from male Jax or Tac mice, and C. after FMT from male Tac or Fast Jax mice. (PDF 764 kb)
