# Supplementary figures and images for: Gut microbes contribute to variation in solid organ transplant outcomes in mice
Source: Microbiome. 2018 May 25;6:96. doi: 10.1186/s40168-018-0474-8 (PMC5968713; doi:10.1186/s40168-018-0474-8)

A

## Shannon Entropy

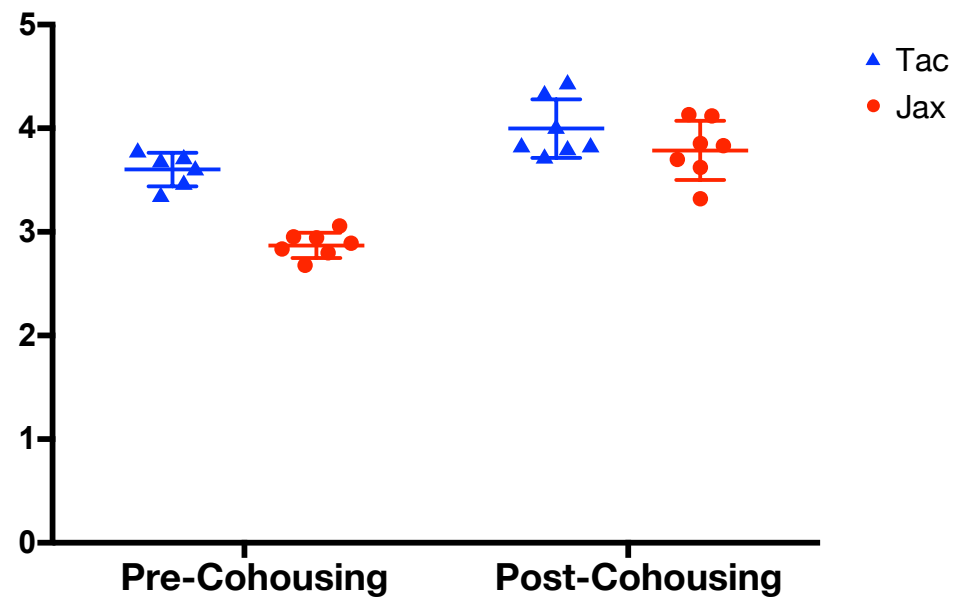

B

## Shannon Entropy

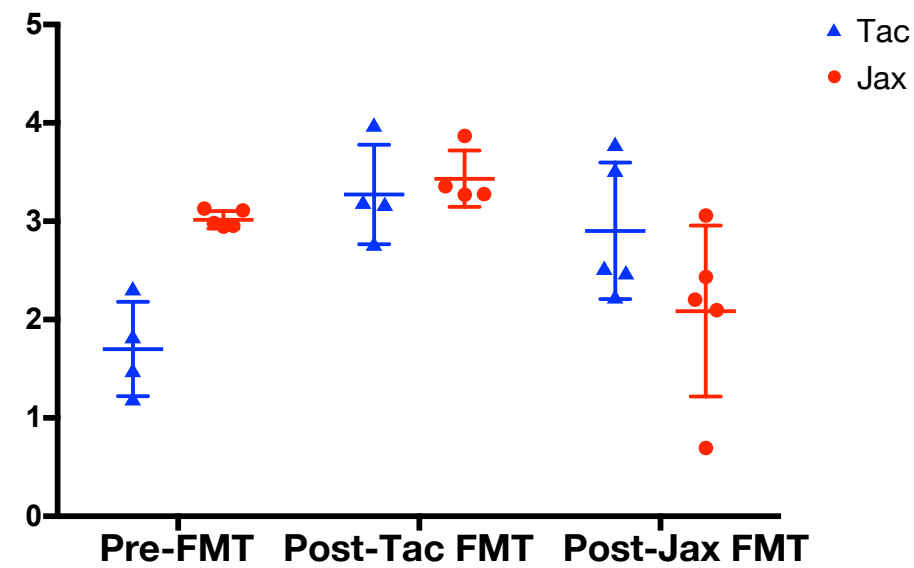

## Inverse Simpson Index

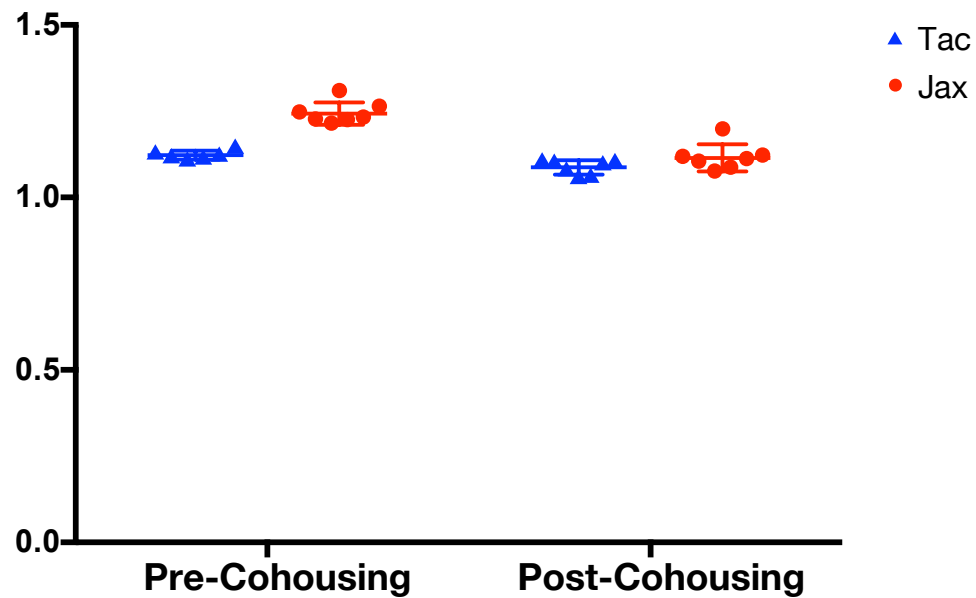

## Inverse Simpson Index

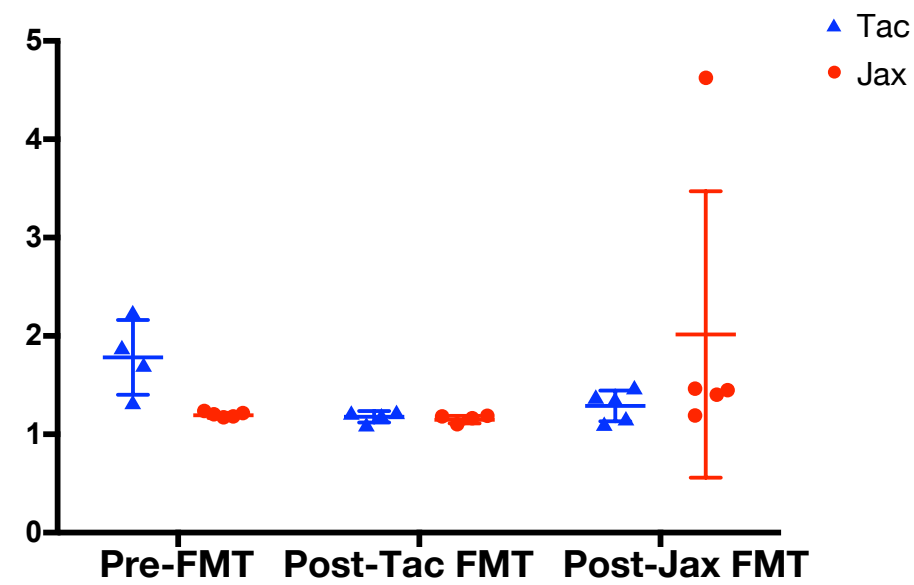

Supplement: Supplementary file 4 — Alpha diversity of the fecal microbiota is not associated with prolonged skin transplant survival in Jax mice. Figure showing Shannon entropy and inverse Simpson index of fecal microbiota from female Jax and Tac skin transplant recipients A. before and after cohousing, and B. before and after FMT from male Jax or Tac mice. (PDF 788 kb) [file 40168_2018_474_MOESM4_ESM.pdf]

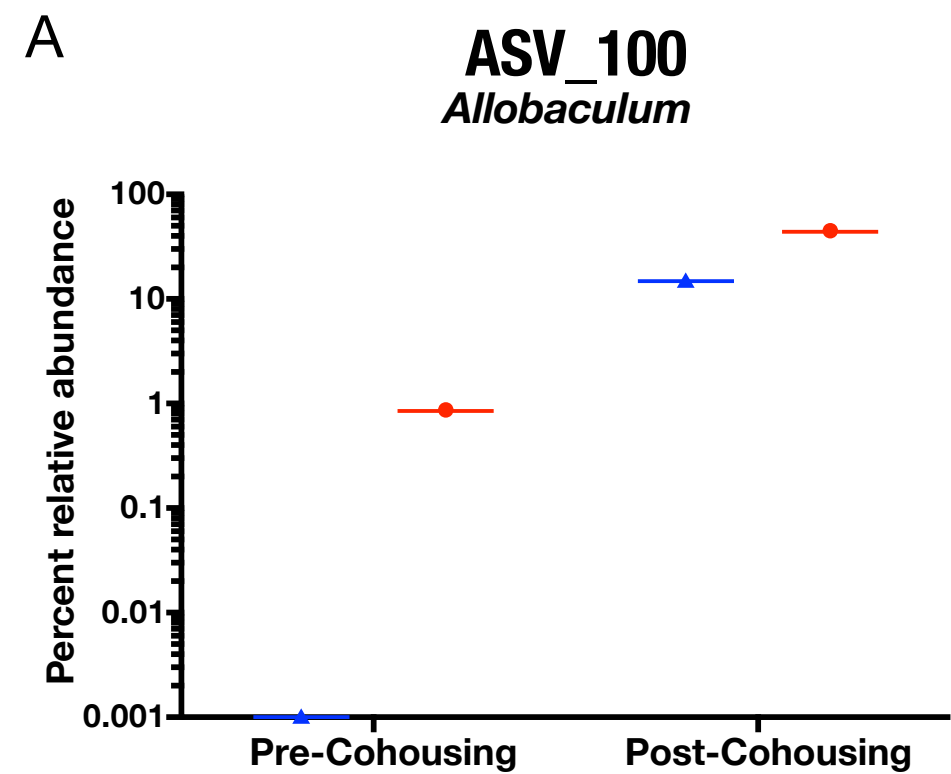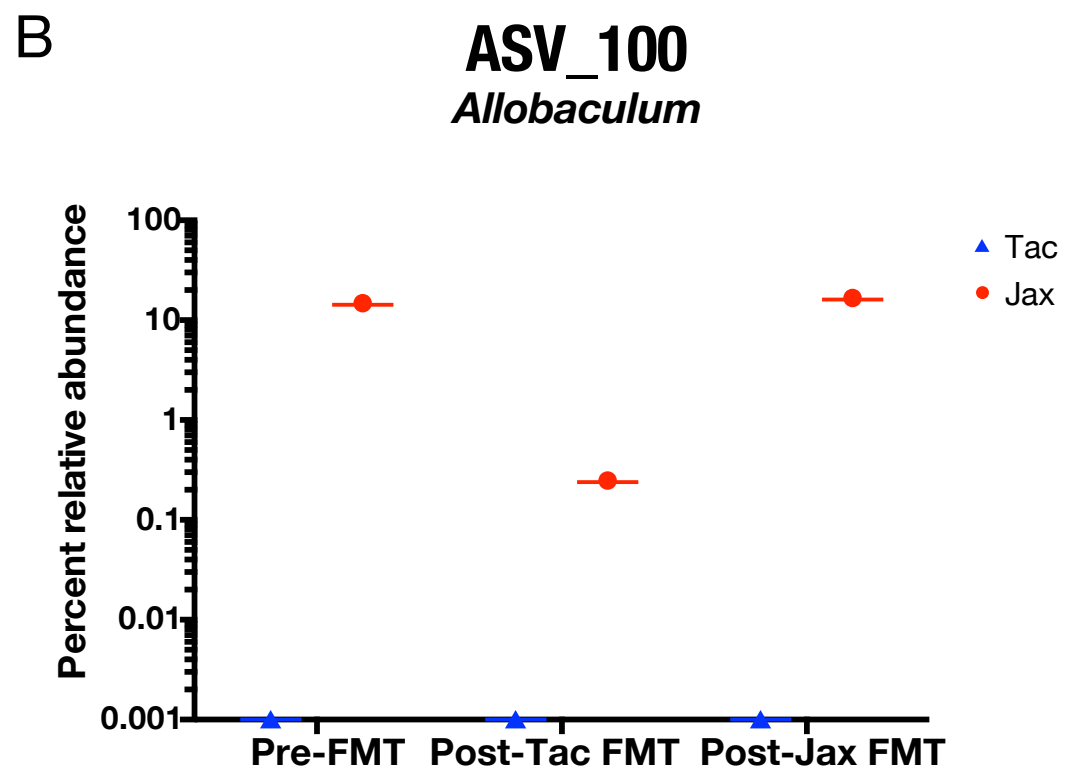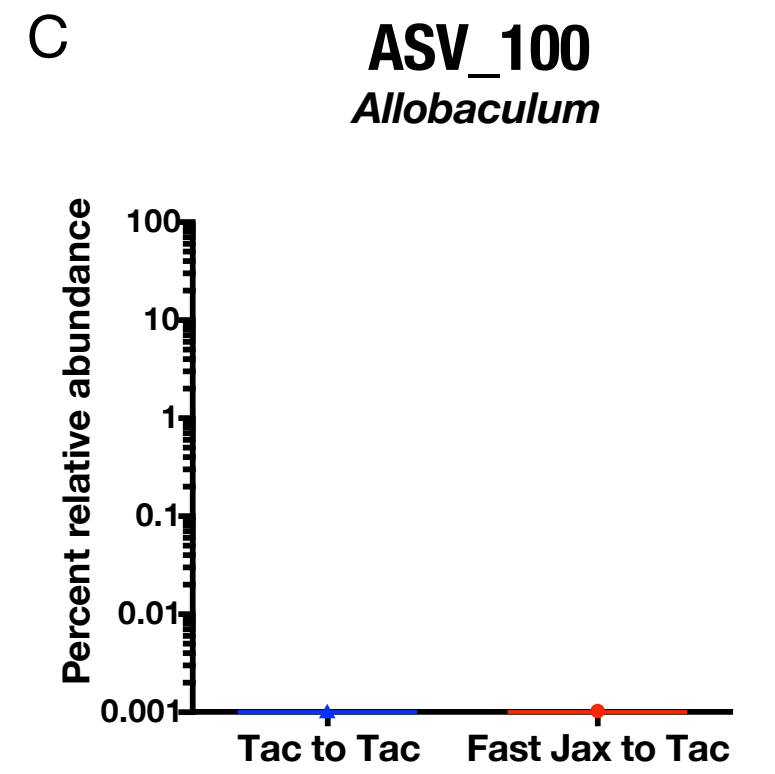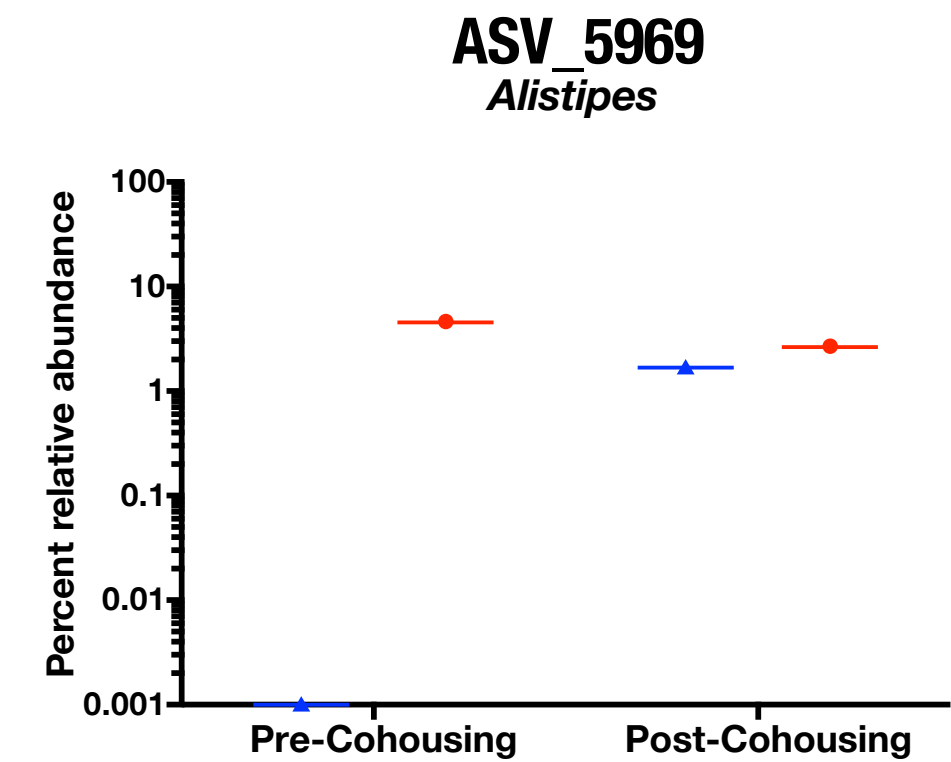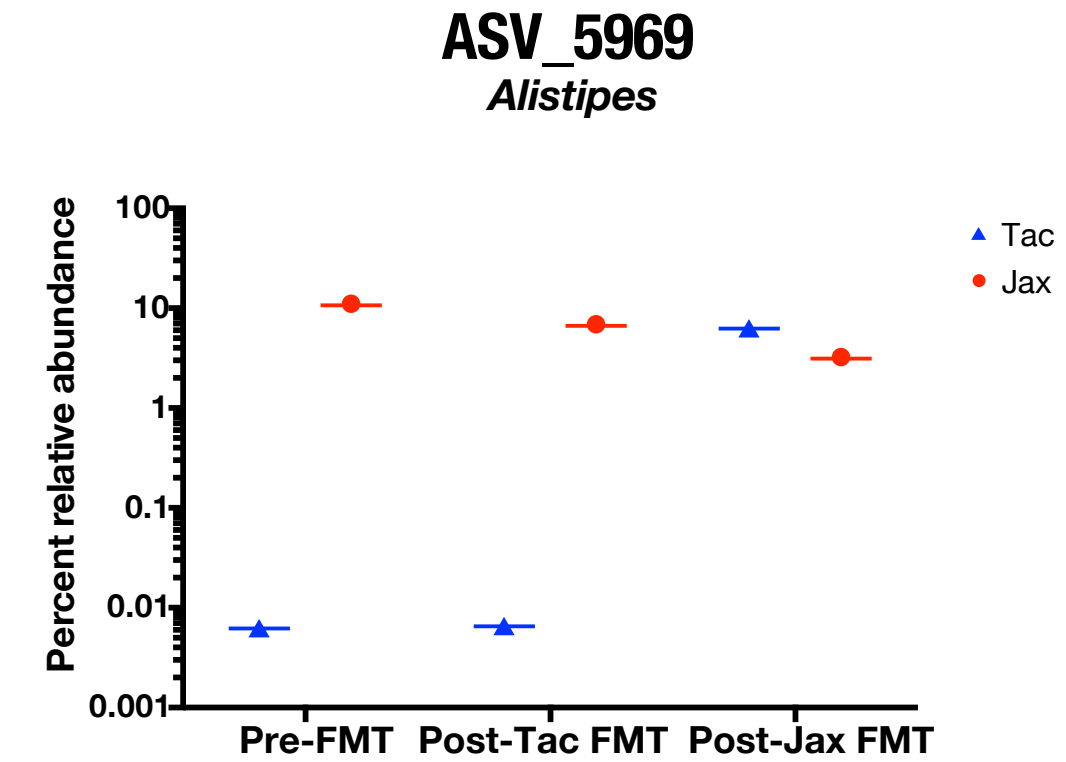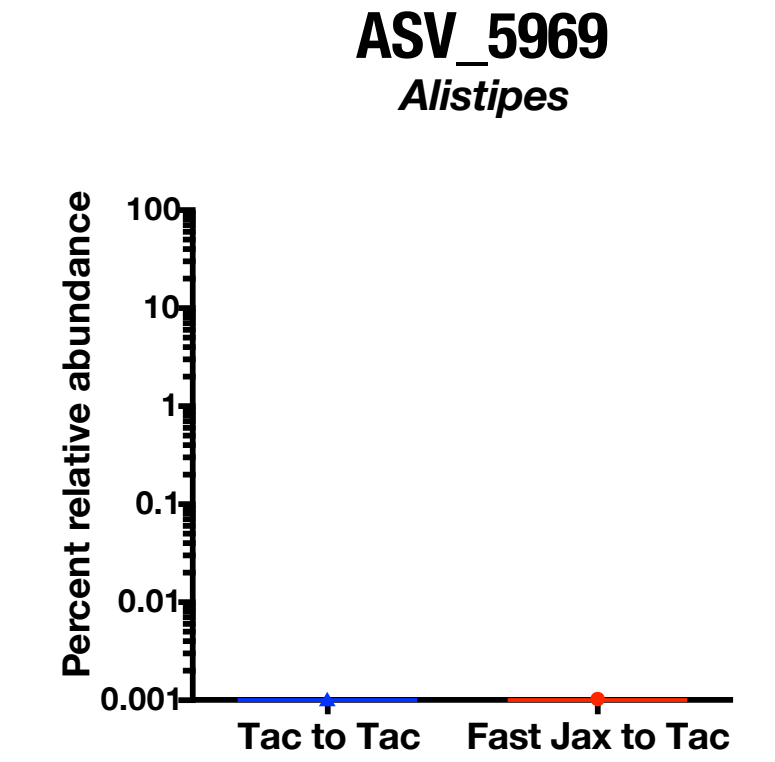

Supplement: Supplementary file 10 — Relative abundance of ASV_5969 in skin transplant donors is associated with prolonged skin transplant survival. Figure showing percent relative abundance of transplant survival-associated ASVs in male Jax and Tac skin transplant donors A. before and after cohousing, B. before and after FMT from male Jax or Tac mice, and C. after FMT from male Tac or Fast Jax mice. (PDF 764 kb) [file 40168_2018_474_MOESM10_ESM.pdf]
